# To Our Subscribers

**Published:** 1871-06

**Authors:** 


### *To our Subscribers.*

We feel that an apology is due our subscribers for the long delay in the appearance of this number of the Journal. We can only say that a combination of circumstanaes, unnecessary to mention here, and which we promise shall not again occur, has caused this delay. The July and August numbers will appear early in August, under the same cover. After that time, the Journal will be issued regularly, the first of each month.

